# Deep-Seated Large Myositis Ossificans: A Diagnostic Blind Spot and the Importance of Early Ultrasonographic Screening

**DOI:** 10.7759/cureus.109853

**Published:** 2026-05-28

**Authors:** Okan Can Karadeniz, Muhammed Yusuf Afacan, Furkan Özönder, Ali Can Baris, Esra Çirci

**Affiliations:** 1 Department of Orthopaedics and Traumatology, Istanbul Physical Therapy and Rehabilitation Training and Research Hospital, Istanbul, TUR; 2 Department of Anatomy, Institute of Graduate Studies, Istanbul University-Cerrahpasa, Cerrahpasa Faculty of Medicine, Istanbul, TUR

**Keywords:** case report, heterotopic ossification, lower extremity trauma, myositis ossificans, radiotherapy (rt), sports injury, thigh contusion, ultrasonography, vastus intermedius

## Abstract

Myositis ossificans (MO) is a benign heterotopic ossification of skeletal muscle, typically resulting from direct trauma. Diagnosing MO confined to deep muscle groups, such as the *vastus intermedius*, can be challenging, often leading to delayed treatment. We present the case of a 39-year-old amateur athlete who developed a giant MO lesion (10x3 cm) two years following a severe thigh contusion. The patient presented with persistent restriction of hip flexion that outlasted the acute inflammatory phase, while a palpable mass was not identified until the chronic stage, 16 months post-injury, due to the deep anatomical localization. Surgical excision was performed, followed by progressive functional recovery. Passive hip flexion improved to 120° by the fourth postoperative week with only minimal end-range pain, and full painless active hip flexion was observed by the eighth week. At the 12-week follow-up, the patient had resumed straight-line jogging without clinical evidence of recurrence, although intermittent anterior thigh cramps persisted during high-intensity exercise. This case highlights the potential pitfalls of relying solely on physical palpation for deep muscle injuries. In such scenarios, early utilization of ultrasonography (US) may be beneficial for the timely detection of MO, as sonographic findings often precede radiographic evidence. Specifically, US typically reveals a hypoechoic mass in the acute phase, which evolves into a hypoechoic lesion containing hyperechoic foci during the intermediate and late stages of maturation. Early detection may help limit lesion progression and reduce the extent of subsequent surgical morbidity.

## Introduction

Myositis ossificans (MO) is a condition that typically occurs in the extremities following trauma [1-3]. More specifically, it is defined as a benign, self-limiting heterotopic ossification of skeletal muscle [4]. MO is commonly categorized into progressive and traumatic forms, with myositis ossificans traumatica representing the typical post-injury subtype [1].

While MO predominantly occurs following trauma (75%), it can also be observed following burns, poliomyelitis, or paraplegia [3,5]. In post-traumatic cases, the most frequently affected muscles are the quadriceps femoris muscle, the adductor muscle group, and the m. brachialis [6-8]. MO may manifest following direct trauma or as a result of repetitive minor injuries [8,9]. The exact mechanism of MO formation remains incompletely understood. In simple terms, it is considered an abnormal healing response in which trauma-induced bleeding and inflammation within the muscle stimulate local fibroblastic or mesenchymal cells to form bone within soft tissue. Osteogenic signaling pathways involving BMP-2, BMP-4, and TGF-β are thought to contribute to this process [2,7,10].

Although post-traumatic myositis ossificans is well described, lesions arising within deep muscle compartments may remain difficult to detect when no superficial mass is palpable. This case report describes a large post-traumatic MO lesion measuring 10 × 3 cm, confined to the vastus intermedius muscle and developing over a two-year period after a football-related thigh contusion. We aim to highlight the diagnostic limitations of physical examination in deep muscle injuries and emphasize the potential role of early ultrasonographic assessment in patients with persistent pain or restricted range of motion after deep muscle contusion.

## Case presentation

A 39-year-old man presented with chronic right thigh pain and a palpable mass following direct trauma sustained during a football match two years earlier. Despite initial conservative management with the RICE (rest, ice, compression, and elevation) protocol, he reported persistent ecchymosis along the midline of the thigh and pain exacerbated by hip flexion and knee extension. Although the acute pain subsided within three months after the injury, functional limitation persisted. Approximately 16 months after the incident, or eight months before presentation, the patient noticed induration in the mid-thigh region. He had no history of spinal cord injury or intensive care unit admission. No ultrasonographic examination was performed at the initial presentation or during the follow-up period. In the present case, early ultrasonographic evaluation would not necessarily have changed the initial conservative management, as early post-contusion MO is generally treated nonoperatively. However, it could have prompted closer clinical surveillance, earlier activity modification, and repeat imaging in the presence of persistent range-of-motion limitation. Earlier recognition of progressive ossification may have allowed conservative management during the maturation phase or, if surgery ultimately became necessary, excision of a smaller lesion with less disruption of the vastus intermedius muscle. 

**Figure 1:**
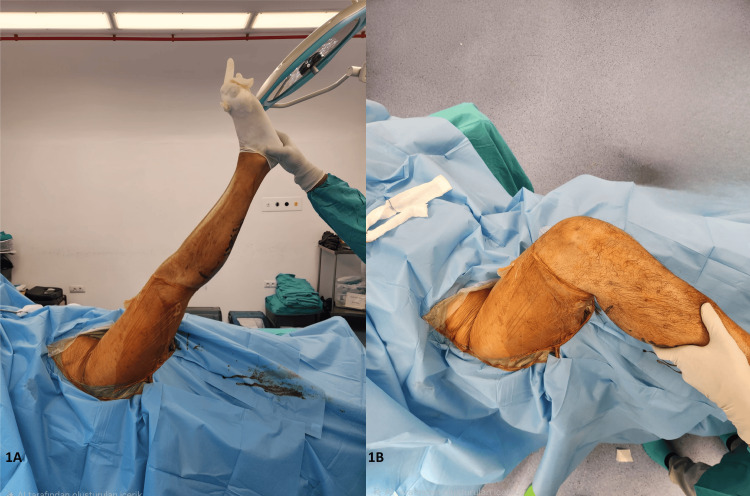
Preoperative clinical assessment of hip flexion. (A) Passive hip flexion with the knee extended (B) With the knee flexed

**Figure 2:**
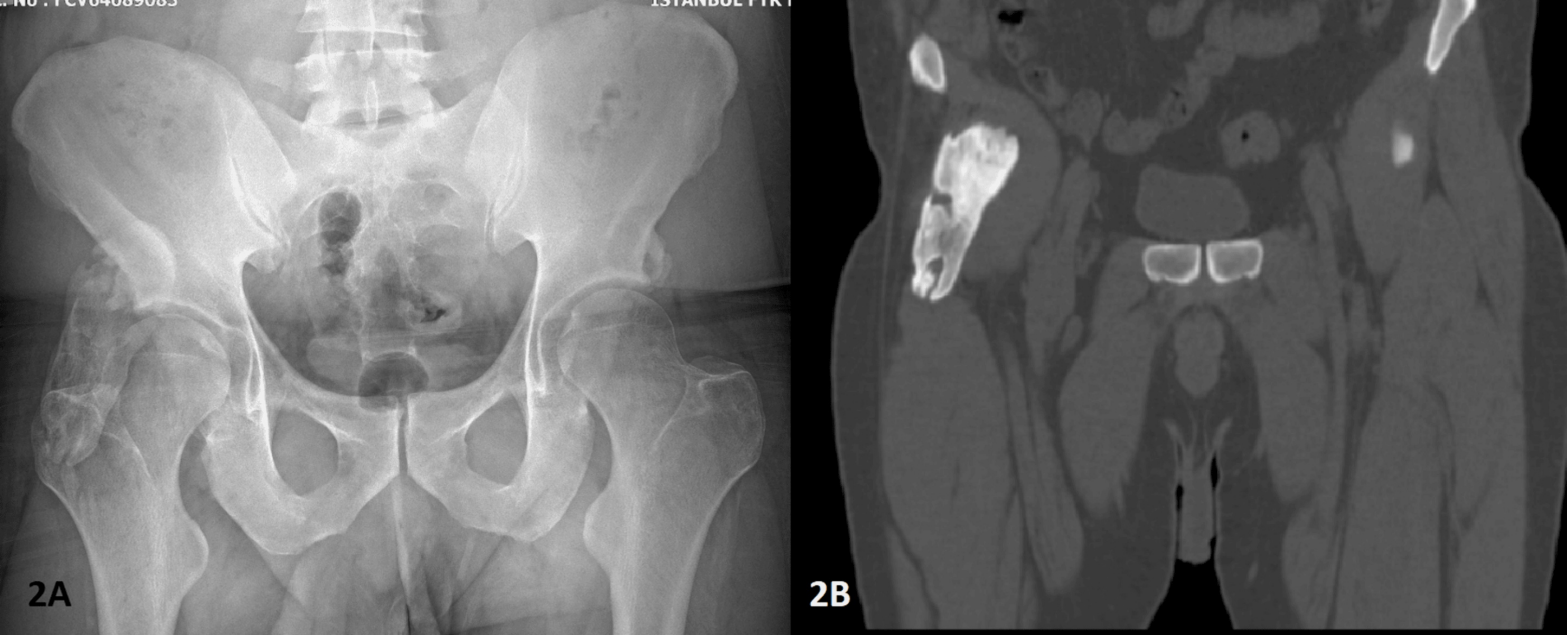
Radiographic and computed tomography findings of the lesion. (A) Plain radiograph showing heterotopic ossification. (B) Coronal computed tomography image demonstrating an ossified mass.

Physical examination revealed painful limitation of hip flexion, restricted to 45° with the knee extended and 75° with the knee flexed (Figures 1A, 1B). The log roll test was negative, although mild pain was elicited during internal and external rotation of the hip. Plain radiographs demonstrated a hyperdense lesion in the right thigh (Figure 2A). Subsequent computed tomography (CT) confirmed a hyperdense mass measuring approximately 10 × 3 cm, located centrally within the vastus intermedius muscle (Figure 2B). These findings were consistent with myositis ossificans.

**Figure 3:**
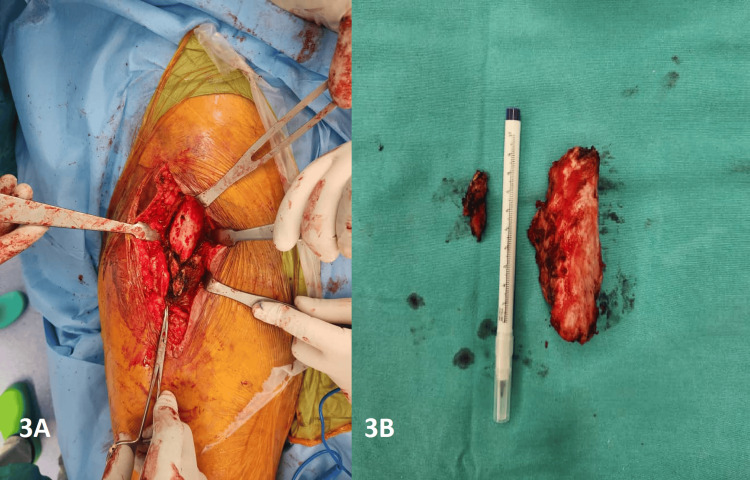
Intraoperative appearance and gross specimen. (A) Intraoperative view of the ossified mass within the vastus intermedius after surgical exposure. (B) Gross appearance of the excised specimen measuring approximately 10 × 3 cm.

**Figure 4:**
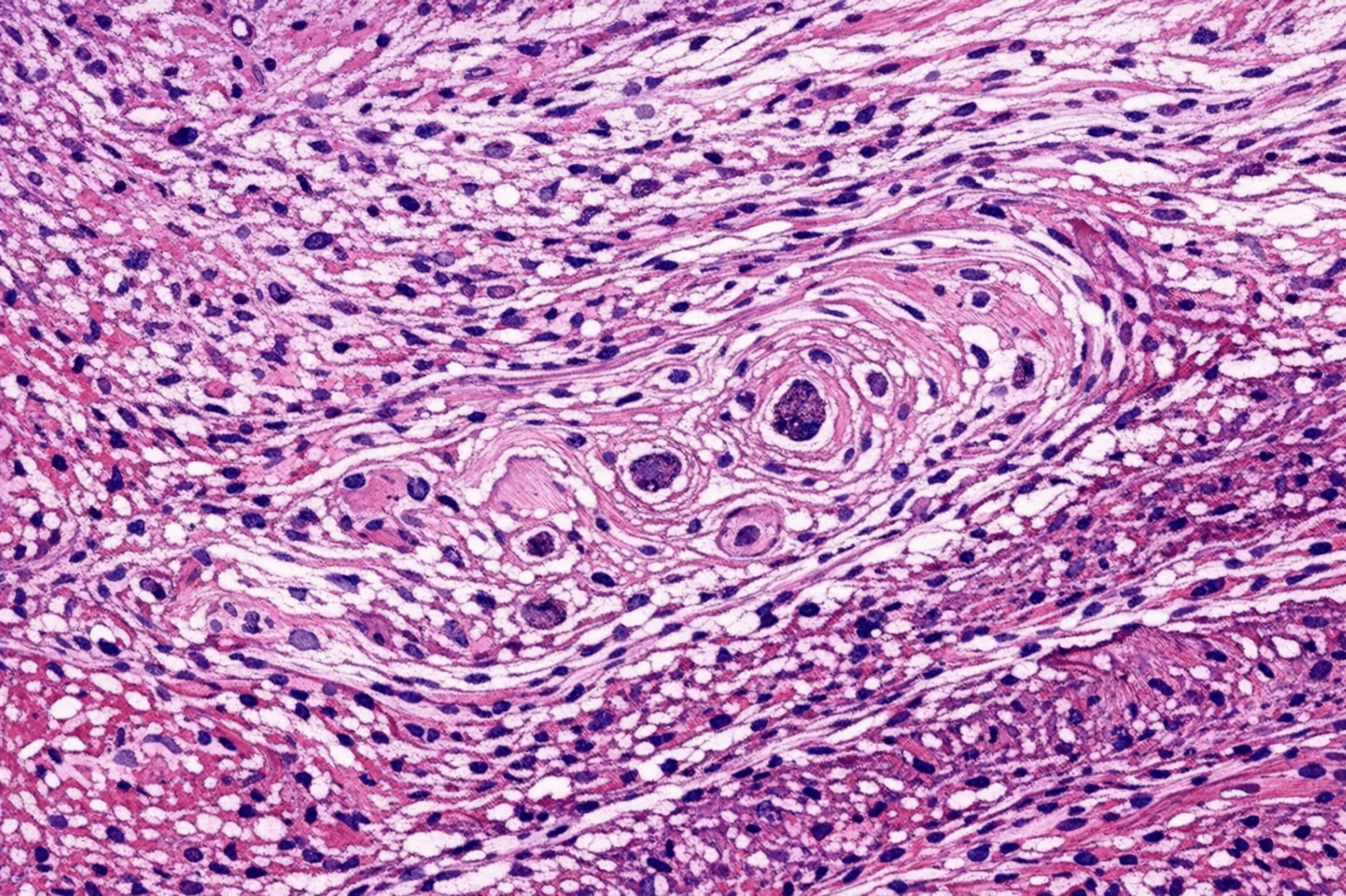
Histopathological appearance of myositis ossificans. Histopathological evaluation of the excised specimen revealed mature lamellar bone formation arranged in a zonal pattern, confirming the diagnosis of myositis ossificans.

Surgical intervention was performed under spinal anesthesia. A standard Smith-Petersen anterior approach was utilized to expose the surgical field [11]. Upon dissection, the ossified mass was identified within the central substance of the vastus intermedius and was carefully excised in its entirety (Figures 3A, 3B). Following excision, the residual muscle fibers on the medial and lateral aspects were primarily repaired to restore continuity without the need for tendon graft reconstruction. A significant increase in hip flexion range of motion (ROM) was confirmed under anesthesia immediately following the procedure. A closed-suction drain was placed, and anatomical closure was performed. Histopathological examination of the excised specimen confirmed the diagnosis of myositis ossificans, demonstrating mature lamellar bone formation with zonal architecture and no evidence of malignancy (Figure 4).

Postoperative prophylaxis against heterotopic ossification recurrence included a single fraction of 700 cGy (7 Gy) external beam radiotherapy (EBRT) using 6 MeV electrons, administered at 24 hours postoperatively. Concurrently, oral indomethacin (25 mg three times daily) was initiated on the first postoperative day and continued for three weeks. Sutures were removed at two weeks postoperatively. By the fourth postoperative week, the patient had achieved 120° of passive hip flexion with minimal end-range pain. At the eight-week follow-up, full and painless active hip flexion was observed. Straight-line jogging was initiated at 12 weeks postoperatively. Although functional recovery was satisfactory, the patient reported intermittent muscle cramps in the anterior thigh during high-intensity exercise.

## Discussion

Myositis ossificans is a benign heterotopic ossification of skeletal muscle that usually develops after acute or repetitive trauma [3]. It is most commonly observed in young adults, particularly during the second and third decades of life, and has a higher prevalence in males [2,12]. Although it typically becomes clinically apparent between the fourth and twelfth weeks after injury, delayed presentations have also been reported in the literature [4,12].

Particularly in athletes, post-traumatic MO formation affects performance and may necessitate surgical intervention in those who do not respond to conservative treatment [2]. Return to sport following surgery is rapid, and patients are often able to ambulate as tolerated without the need for a brace or walker post-operatively [12]. Elite and professional athletes are often managed within structured medical and rehabilitation systems after injury, whereas similar follow-up pathways may be less consistently available for amateur or recreational athletes. This distinction is clinically relevant, as previous football injury data have suggested lower recurrence rates at the highest professional level compared with national and amateur levels, potentially reflecting differences in sports medicine and physiotherapy support [13].

In the case presented, the patient received a severe blow to the thigh region during a football match two years before presentation. In the acute phase, the patient, who presented with intense pain, limited hip flexion, and ecchymosis, was managed with conservative treatment in the outpatient setting. During this period, only plain radiography was performed, and no pathology was detected. However, as frequently noted in the literature, plain radiographs may not reveal any findings following trauma, whereas US examinations may demonstrate focal hypoechoic areas [14]. These findings suggest that ultrasonography may have a role in the follow-up of patients with persistent symptoms after deep muscle contusion.

The patient presented to our clinic 24 months after the trauma and eight months after his complaints intensified. Imaging revealed ossified tissue measuring 10x3 cm. The ossified tissue affected the majority of the vastus intermedius and left a defect following surgery. In such scenarios, early diagnosis via ultrasonography may allow timely conservative management and potentially reduce progression, such as weight-bearing restrictions, rest, passive stretching, cryotherapy, and elevation, given the self-limiting nature of the disease. Furthermore, even in cases necessitating surgical intervention, early sonographic detection facilitates the excision of the lesion while it is still diminutive, thereby preserving soft tissue and muscle integrity and minimizing the functional impact on the athlete.

## Conclusions

In conclusion, myositis ossificans arising within deep muscle compartments, such as the vastus intermedius, may remain clinically unrecognized for a prolonged period because physical examination and plain radiography can be insufficient in the early stages. Persistent pain or restricted range of motion after deep muscle contusion should prompt consideration of early ultrasonographic evaluation, even when no palpable mass is present. Earlier recognition may allow closer follow-up, timely conservative management, or, when surgery is required, less extensive excision with better preservation of muscle integrity. Although this single case cannot establish a definitive preventive role for ultrasonography, it highlights its potential value as an adjunctive tool in the evaluation of persistent symptoms after deep muscle trauma.

## References

[1] Hanisch M, Hanisch L, Fröhlich LF (2018). Myositis ossificans traumatica of the masticatory muscles: etiology, diagnosis and treatment. Head Face Med.

[2] Devilbiss Z, Hess M, Ho GW (2018). Myositis ossificans in sport: a review. Curr Sports Med Rep.

[3] Savvidou O, Papakonstantinou O, Lakiotaki E (2021). Post-traumatic myositis ossificans: a benign lesion that simulates malignant bone and soft tissue tumours. EFORT Open Rev.

[4] Saad A, Azzopardi C, Patel A (2021). Myositis ossificans revisited - the largest reported case series. J Clin Orthop Trauma.

[5] Tyler P, Saifuddin A (2010). The imaging of myositis ossificans. Semin Musculoskelet Radiol.

[6] Ngai A (2017). Post-Traumatic Myositis Ossificans.

[7] Kalebić P, Šegulja S, Miletić B (2025). Early surgical treatment of posttraumatic myositis ossificans of the vastus intermedius muscle. J Sport Rehabil.

[8] Buselli P, Coco V, Notarnicola A (2010). Shock waves in the treatment of post-traumatic myositis ossificans. Ultrasound Med Biol.

[9] Walczak BE, Johnson CN, Howe BM (2015). Myositis ossificans. J Am Acad Orthop Surg.

[10] Nuovo MA, Norman A, Chumas J, Ackerman LV (1992). Myositis ossificans with atypical clinical, radiographic, or pathologic findings: a review of 23 cases. Skeletal Radiol.

[11] Smith-Petersen Smith-Petersen, M. N. (1917). A new supra-articular subperiostal approach to the hip joint. Amr Jr Ortho Sur.

[12] Orava S, Sinikumpu JJ, Sarimo J (2017). Surgical excision of symptomatic mature posttraumatic myositis ossificans: characteristics and outcomes in 32 athletes. Knee Surg Sports Traumatol Arthrosc.

[13] Hägglund M, Waldén M, Ekstrand J (2016). Injury recurrence is lower at the highest professional football level than at national and amateur levels: does sports medicine and sports physiotherapy deliver?. Br J Sports Med.

[14] Cren PY, Penel N, Cordoba A (2022). Myositis ossificans circumscripta after surgery and radiotherapy and during sunitinib treatment: a case report. J Med Case Rep.

